# Requirements for Hybrid Technology Enabling the Production of High-Precision Thin-Wall Castings

**DOI:** 10.3390/ma15113805

**Published:** 2022-05-26

**Authors:** Vladimír Krutiš, Pavel Novosad, Antonín Záděra, Václav Kaňa

**Affiliations:** 1 Institute of Manufacturing Technology, Brno University of Technology, 616 69 Brno, Czech Republic; zadera@fme.vutbr.cz (A.Z.); kana@fme.vutbr.cz (V.K.); 2 Institute of Machine and Industrial Design, Brno University of Technology, 616 69 Brno, Czech Republic; 208950@vutbr.cz

**Keywords:** topological optimization, hybrid technology, additive manufacturing, investment casting

## Abstract

Prototypes and small series production of metal thin-walled components is a field for the use of a number of additive technologies. This method has certain limits related to the size and price of the parts, productivity, or the type of requested material. On the other hand, conventional production methods encounter the limits of shape, which are currently associated with the implementation of optimization methods such as topological optimization or generative design. An effective solution is employing hybrid technology, which combines the advantages of 3D model printing and conventional casting production methods. This paper describes the design of aluminum casting using topological optimization and technological co-design for the purpose of switching to new manufacturing technology. It characterizes the requirements of hybrid technology for the material and properties of the model in relation to the production operations of the investment casting technology. Optical roughness measurement compares the surface quality in a standard wax model and a model obtained by additive manufacturing (AM) of polymethyl methacrylate (PMMA) using the binder jetting method. The surface quality results of the 3D printed model evaluated by measuring the surface roughness are lower than for the standard wax model; however, they still meet the requirements of prototype production technology. The measurements proved that the PMMA model has half the thermal expansion in the measured interval compared to the wax model, which was confirmed by minimal shape deviations in the dimensional analysis.

## 1. Introduction

The requirements for the production of thin-wall castings (TWC) [1] are based on considerable pressure to decrease the weight of the parts produced, especially in the automobile and aviation industries. They are connected with the expression “light-weighting”, which is not only a trend of replacing steel castings by aluminum alloys, but it also involves designing a part with the aim of finding a compromise between the design, manufacturability, properties, and the price of the part being produced [2]. This is why the requirements are increasing for the almost unlimited shape variability of the castings, thinner walls, and higher mechanical properties of the parts, which are connected with the internal and surface quality. Moreover, there is a growing demand for the fast supply of the first prototypes and verification series. Therefore, many foundries are forced to implement rapid prototyping technologies in order to comply with the new trends and technologies. Additive manufacturing (AM) development raises possibilities for foundry technology. Using these new methods requires the implementation of virtual engineering [3] and optimization methods in the initial development phases so that, if possible, the casting can be cast at the first attempt.

Recently, the shapes of castings have been designed to meet at least the basic requirements of the construction technology (DFM—design for manufacturability). It is the adaptation of the part construction to the production method to ensure efficient and quality production [4]. The construction technology was focused on the model partition with respect to the pre-selected production technology focused on the elimination of hot spots by an ideal connection and wall transition to the elimination of foundry defects. For these reasons, the shapes of the castings were rather conservative to meet the requirements for functionality and production technology. The development of new optimization methods in 3D design such as topological optimization or generative design and the development of 3D printing methods have shifted the design of part shapes to an area that previously only belonged to artistic castings.

The investment casting (IC) technology complies both with the specific requirements for casting thin-wall castings and the wide material variability and possibility to produce complex shapes. It is also a technology that is widely used for producing prototypes and can be well combined with the AM (in the sequel referred to as 3D printing) of models, ceramic cores or shells. This combination is called “hybrid technology” [5].

The main way of using hybrid technology in IC is the 3D printing of models, subsequent production of a ceramic shell, and its casting in a conventional way. An overview of the use of rapid prototyping (RP) for thin-walled castings, including individual methods, is published in the review [6]. The advantages of the hybrid technology of 3D printed model production lie mainly in low costs and time savings for prototype or small series production and the possibility of casting complex shapes, which are difficult to produce in a conventional way. Hybrid technology is in a sense a competitor to direct metal printing and the choice for this technology will depend mainly on the shape of the part and the quantity required [7]. The use of rapid prototyping in model printing was first mentioned in 1989 [8]. These are methods based on stereolithography (SLA). Mukhtarkhanov et al. (2020) enumerated the history, development, advantages, and disadvantages of this method for model production [9]. A far cheaper method exploited widely by engineers today is material extrusion AM-fused deposition modelling (FDM) [10] or fused filament fabrication (FFF) method, which uses plastics such as ABS or PLA to print models [11]. A disadvantage of the FDM and FFF methods is low surface quality of the models. Methods of solving the stair-stepping structure are dealt with, for example in [12]. Whether it is the SLA or FDM method, the main problem when using these methods and materials is the cracking of the ceramic shells in the melting and firing phase. As described in [10,13,14], this phenomenon is associated with the expansion of the material used or the construction and shape of the model infill. For this reason, materials are being developed that have low thermal expansion or that soften when certain temperatures are reached [15]. Polymethyl methacrylate (PMMA) in powder form seems to be an interesting material in terms of low thermal expansion, which is used in the binder jetting (BJ) technology [16] while maintaining surface quality, low ash content, and a high level of dimensional accuracy [17]. There is a lack of information available on this material and the BJ technology; therefore, this work partly deals with the measurement of material characteristics.

## 2. Materials and Methods

### 2.1. Topology Casting Optimization

**Figure 1:**
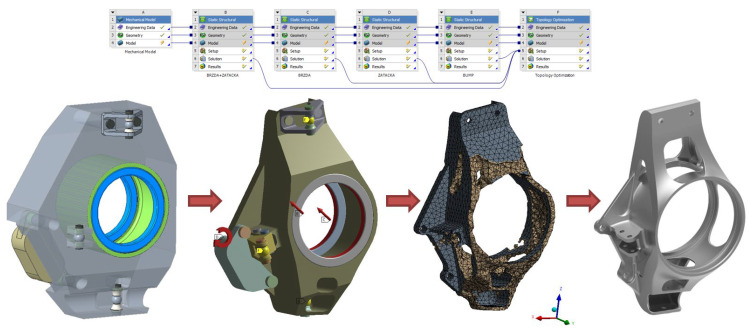
Topological optimization workflow.

The project is based on the requirements set out by a team of Formula Student designers from Brno University of Technology participating in a prestigious European competition among university teams, in which the aim is to build a single-seat racing car that must be easily controllable, powerful, reliable, and safe at the same time. The project focused on the possibility to change the production technology from a machined part to a casting, specifically for a new Formula concept designated as Dragon X. The main aim was to design a casting that would be lighter while maintaining the stiffness, would have smaller deformations, and its production would not be lengthy and costly. The part in question is an upright, which is one of the most important parts of the suspension system. The task of the uprights is to transfer the load from the driving forces to the vehicle suspension, to mount the brake caliper and, in the case of a steered axle, also the steering point. Considering the fact that it is unsprung mass, it is important to keep the weight of the upright low. However, it should not be implemented at the expense of decreasing the stiffness of the whole mounting, which is the most important factor in the design. If the bearing of the wheel is too flexible, it may distort the driving experience and the response of the whole car, which then becomes unpredictable and difficult to control for the driver [6]. When producing the design, topological optimization of the part was first performed by the ANSYS Workbench 18.1, which became an inspiration for the design and helped to improve the resulting parameters of the part. Subsequently, the modelling of the upright started in the SolidWorks 3D. Figure 1 shows an optimization chain procedure containing defined load conditions and a limit for material preservation being 20 % of the original volume. 

The aluminum alloy EN AC-AlSi7Mg0.3 was chosen as the material for the future casting, considering the heat treatment T6 to increase the mechanical properties. FEM component analyses were performed during the topological optimization and for the final design. The upright design was simulated using the finite element method in the ANSYS Mechanical software and structural static calculations were performed.

**Figure 2:**
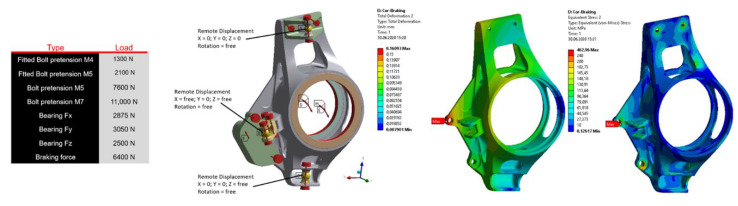
Assessment of the optimized casting shape for the stress conditions of turning and braking (distribution of deformation and equivalent stress).

**Figure 3:**
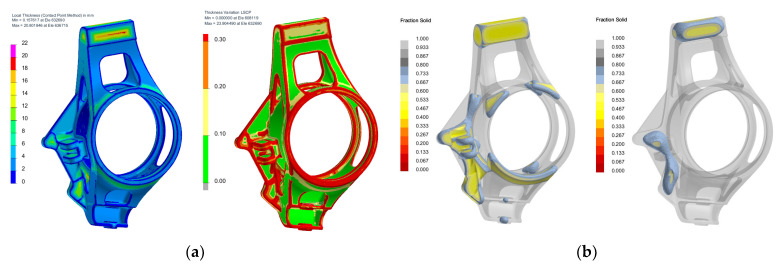
Co-design results. (**a**) Wall thickness analysis; (**b**) Simulation of hot spot formation.

The assessment criteria were strength and stiffness of the mounting and safety related to the ultimate limit of stress and strain, which was required to be at least 1.2, which corresponds to a maximum reduced stress of 200 MPa. An example of the FEM analysis for the stress conditions of turning and braking is shown in Figure 2. The casting design of an upright for the Dragon X vehicle had better maximum deformation results in all the stress conditions. Compared to the previous generation, the final weight of the part decreased by 12% while the stiffness increased by 25%. The final external dimensions of the upright are 150 × 80 × 40 mm. The optimized geometry was then explored with regard to manufacturability in a special module of the ProCAST software called Co-design. This approach enabled the verification of the manufacturability of the part with respect to the selected production technology (DFM) focusing on the wall thickness transitions, radii of the part, and occurrence of hot spots—Figure 3. Inappropriate selection of wall transitions, sharpness of angles, and size of the radii may lead to the formation of hot spots in the casting or formation of hot tears or cracks. Based on the analysis, the radii around the base were modified (increasing the radius from 1 mm to 2 mm) and in the center part of the hub the lightening holes were modified in order to make the subsequent drying of the ceramic shell easier. The last sections of the casting solidification were selected as sections for attaching the gating system so that, through these connections, metal could be fed from the gating system during the solidification. The castings were also subjected to a numerical simulation of the ceramic shell pre-heating, metal filling and solidification including a prediction of the occurrence of defects such as shrinkage cavities and porosities.

**Figure 4:**
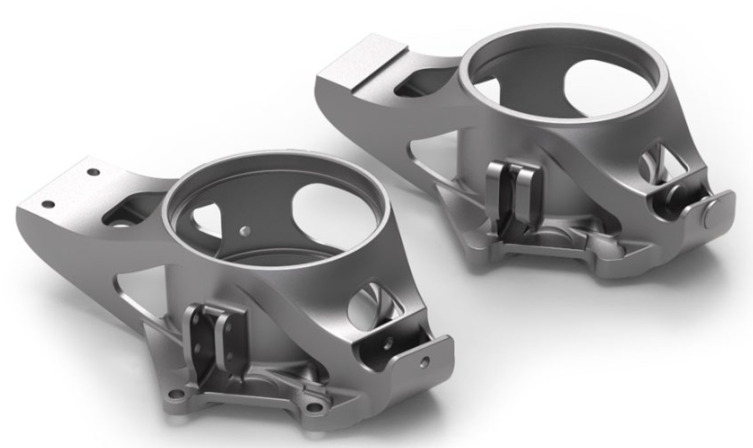
The final 3D data of the workpiece (**left**) and casting (**right**).

The casting model was finally modified with machining allowances, it was enlarged by 1.2% for the shrinkage of the aluminum alloy, the functional surfaces were supplied with machining allowances, and small holes for anchoring the part were sealed—Figure 4.

**Figure 5:**
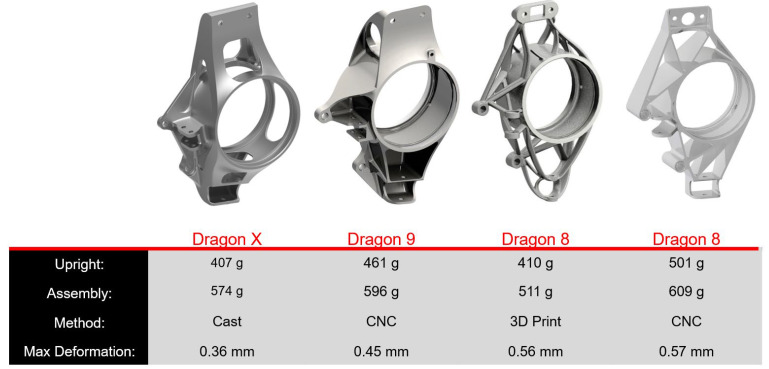
Comparison of parameters of the different Formula Student versions.

Figure 5 maps the development of the shape and production technology used for the upright, specifically from the Dragon 8 Formula to Dragon X. In recent years, the team experienced the production of uprights from aluminum alloy using additive technology for the Dragon 8 single-seater and aluminum milling for Dragon 9. Each technology has its pitfalls and benefits. Production using additive technology gives the designer a free hand in terms of the shape of the part. However, its disadvantage is a complex and expensive production technology, especially for larger parts and a large number of the parts required. During production using milling, the designer must bear in mind the limits of this technology (shape limits) and make sure that the design is machinable. An advantage is the availability of the production equipment. Production using aluminum casting should combine the advantages of both of the previous manufacturing processes and appears to be a promising possibility for future use [17].

### 2.2. Investment Casting Requirements

The investment casting technology is a “net shape” technology. It is regarded as one of the most precise foundry methods in terms of dimension tolerance (DCTG 4-8–DIN EN ISO 8062-3) and it is among the technologies that can produce a high-quality surface in the as-cast condition (Ra 1.6-12.5) [18]. Generally, it is suitable for smaller castings. It is highly effective for prototype parts and thin-wall castings. This method is commonly used to produce small and medium series production castings in the aviation and medical sectors, in transport and power engineering (power generation industries). Compared to conventional foundry methods, complex shapes can be produced that would be difficult to make due to a complex parting line. Introduction of 3D printing pushes this limit even further because, to make the model, there is no need for a metal mold (for wax models), which also has its design limits. 

The following are the specifics of the investment casting technology related to the shape of the part [19].

- Min. radius on the outer and inner edges 1 mm;
- The smallest theoretical wall thickness 0.8 mm (depends on the casting size and shape, material, temperature, etc.) and the common casting thickness is 2–4 mm;
- No draft angle has to be factored;
- Gradual wall transition towards feeding (directed solidification towards the gate);
- Use transition ribs for thin walls (increasing stiffness, metal flow, decreasing deformation);
- If possible, do not close the internal casting cavities (with regard to the drying of the ceramic layers and subsequent removal of the shell);
- With the 3D printed model, model the gates right away (better connection of the part to the gating system).

The shape variability of 3D printing practically has no limits and fully supports modern trends using shape optimization tools such as topological optimization and generative design. However, certain requirements for the model and the 3D print material have to be met when using hybrid technology. The basic requirements for the model include:

- High surface quality of the printed model (connected with the 3D print method and the possible surface finish);
- High dimensional accuracy of the printed model (affects the final dimensions of the casting);
- Low thermal expansion of the material (higher expansion causes the ceramic shell to crack when being fired);
- Low content of ash matter after the model burns (high content of ash matter requires long firing. High ash content may have a negative effect on the resulting surface quality of the casting).

The casting assembly can be printed directly or a combination is used of wax down sprue and fusible connection with the printed model. In some cases, mechanical securing of the junction is necessary to prevent the models from breaking off the gating system. The subsequent dipping of the printed model in ceramic slurry is not different from dipping a wax model. In most cases, the wettability does not have to be modified by using special agents. Stucco coating of the ceramic material and the subsequent drying of the shell layers is the same as in the classic procedures. Unlike the technology that uses a wax model and subsequent de-waxing in a boiler-clave, 3D printed models are fired in annealing furnaces with an afterburning chamber. The afterburning temperature ranges from 600 to 800 °C based on the material of the model printed. What can be problematic is the de-waxing of combined gating systems if sufficient temperature shock is not ensured for the wax model. In that case, the shell may crack during the firing.

## 3. Results

### 3.1. Properties of a Model Made by 3D Printing 

The hybrid casting production technology is based on the 3D printing of a model, which is then used for the production of a ceramic shell. In the last step, this shell is used as a mold for the production of a metal casting. As described above, for the hybrid technology for casting production using investment casting, it is necessary to ensure dimensional accuracy of the model, good surface quality and to use material with low thermal expansion to prevent the occurrence of cracks in the shell during the firing phase.

**Figure 6:**
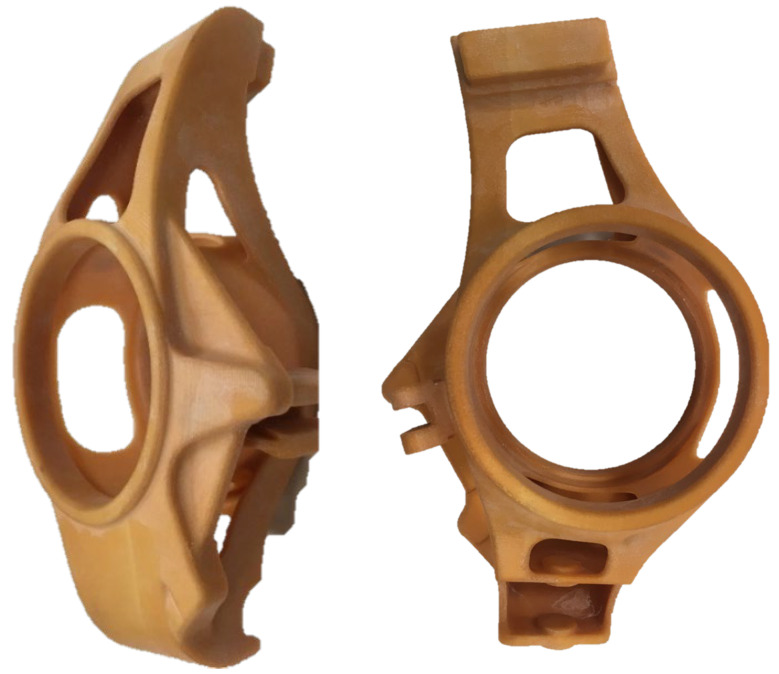
The upright model printed by the BJ (binder jetting) method.

The upright model was 3D printed by the binder jetting (BJ) method using PMMA. Polymethyl methacrylate (PMMA) is an acrylic plastic with excellent burnout behavior. PolyPor B was used as a binder. The printing resolution used was 600 dpi, the thickness of the printed layer was 150 µm, and the final surface of the model was treated using wax infiltration. The printing was performed in cooperation with the Voxeljet company. The part printed is shown in Figure 6.

#### 3.1.1. Assessment of Dimensional Changes in the Model

The investment casting method is directly intended for making “NET SHAPE” castings, when further shaping is not expected; therefore, the casting must already be made in the “as-cast condition” with narrow dimensional tolerances [19]. Knowledge of the behavior of the wax model during its production is, from the perspective of dimensional accuracy of the final casting, only one part of the necessary comprehensive knowledge of dimensional changes during the whole technological flow. This means that knowledge of dimensional changes during the production of the shell (coating, de-waxing, drying, and shell annealing) is also necessary as well as changes after the pouring of the liquid metal and during the solidification and cooling stages. What is not insignificant is the shape factor and constrained shrinkage [20].

When making models using classic wax and a mold, shrinkage is always applied to the model (0.8–1.2%). For printed models, this shrinkage is not commonly applied. Only shrinkage of the cast material or a dimensional change in the shell are considered. Nevertheless, some materials and printing technologies are sensitive to temperature and with larger and less compact shape model deformations may occur. Therefore, it is also necessary to check dimensional deviations from the CAD data.

**Figure 7:**
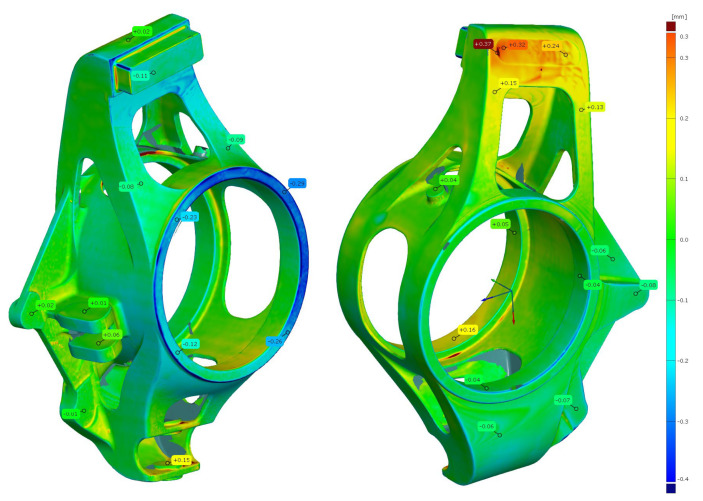
Dimensional changes of printed model (PMMA−BJ method).

Optical measurement of the upright model was carried out by the Atos Core device using the best fit function for model positioning. Figure 7 shows an evaluation of dimensional changes compared with the CAD model.

By the ISO 8062-3 standard, for a nominal dimension of up to 160 mm of a casting made by investment casting, the permitted length dimensional tolerance (DCTG 5) is 0.62 mm. For geometric flatness tolerance (GCTG 5), for example, the maximum permissible tolerance (for dimensions of 100–300 mm) is 1.4 mm. The largest deviation measured on the model was the one in negative direction −0.29 mm, specifically on the upper surface of the central hub. However, in this part, there is a machining allowance of 1.1 mm, so this deviation is not a problem and is compensated for by the allowance. The maximum deviation from the CAD model was +0.37 mm in the recess part. Visually, this deviation looks like a thicker layer after a treatment of the PMMA model by hot wax infiltration. Again, this local spot does not constitute a production problem given the positive deviation and subsequent machining of this spot. The remaining shape deviations were in the range of ±0.2 mm. The dimensional accuracy of the model is thus satisfactory and the model can be used to make the final casting. The accuracy of the 3D printed model is sufficient and all the deviations in the critical parts of the functional surfaces were within the limits.

#### 3.1.2. Assessment of Surface Roughness

**Figure 8:**
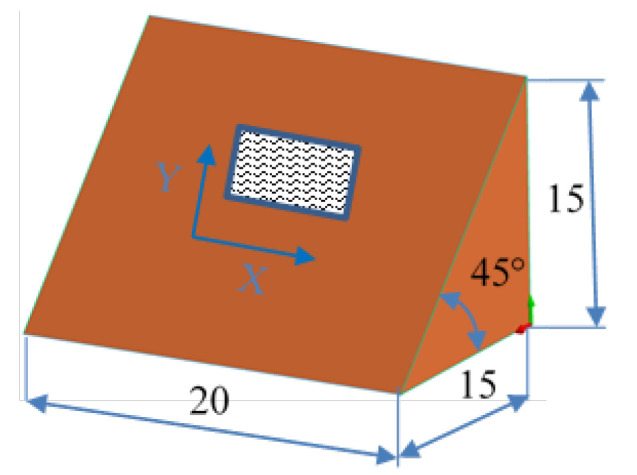
Sample geometry used for the roughness analysis (printing position).

The model roughness was measured on a test body in the shape of a wedge with an angle of 45°—Figure 8. The body was selected this simple so that it would be possible to compare the printed sample (Sample A) with the wax model injected into a metal mold (Sample B). All the roughness measurements were made using the Talysurf CCI Lite device. It is a contactless 3D profilometer based on the principle of coherence correlation interferometry. The device has an image sensor with a resolution of 1024 × 1024 pixels and three Mirau lenses with 10×, 20×, and 50× magnification. Assessed areas sized approximately 1.65 × 1.65 mm, 0.825 × 0.825 mm, and 0.33 × 0.33 mm correspond to these lenses. All the measurements presented below were made using a lens with 20×.

The data obtained by the measurement were then processed in the TalyMap Gold software, which enables the creation of a 2D and 3D model of the analyzed surface. The program uses different ways of model surface treatment such as surface levelling or shape removal, interpolation of unmeasured points, etc., assessment of various surface structure parameters based on a number of standards, and data export in various formats for further processing. Table 1 summarizes the key measurement results. It contains a graphical representation of a 3D model of the sample structure including the *Z* axis, which is given in µm. It can be stated that the structure of the 3D printed sample, compared to the wax sample, is relatively heterogenous with uneven occurrence of recesses and projections. This is related to the technology of 3D print layers of 0.16 mm, which are then partly smoothed out by the final surface treatment—wax infiltration. The table also contains graphical evaluation of the average profile in the *X* axis and *Y* axis directions. The Y direction is transverse to the printing direction. From the entire scanned area, a total of 1024 basic profiles in the transverse direction were created, based on which the roughness height parameters were calculated in compliance with the ISO 4287 standard. Table 1 shows the roughness parameters calculated from all the extracted basic profiles. 

Sample A represents a casting model made by the conventional method of injecting wax into a metal mold. The Ra roughness measured on the model along the *X* axis was 2.68 μm and 2.63 μm along the *Y* axis. The results suggest that the roughness expressed by the Ra parameter both in the transverse and longitudinal direction is comparable. Additionally, the Rz parameter, expressing the average size of the projections and recesses on the surface, is completely comparable in the transverse and longitudinal directions. The average size of the projections on the surface (Rp) is comparable to the average size of the recesses (Rv). Such even surface roughness of the wax model is related to the roughness of the metal mold made by the conventional method of metal cutting. In wax models with similar surface roughness, it is possible to achieve the final Ra roughness of up to 1.6 μm, which can be compared to a conventionally turned or milled surface.

It is clear from the basic profile of Sample B (Table 1—column on the right) made by the 3D print technology that the projections on the sample surface (red color) are not distributed evenly on the surface but in lines corresponding to the motion trajectory of the printhead. More significant differences between the roughness of the conventional wax model and the 3D printed model can be observed on the Rz parameter. The values in Table 1 indicate that the heights of the recesses and projections in the 3D printed model are approx. 4 times greater, and therefore the average value of the Rz parameter is 4 times higher. The Ra roughness parameter for the 3D printed model in the longitudinal X direction is 11.34 μm, and in the transverse Y direction the Ra is 9.09, which is approximately 3 times higher than for the wax model molded in the metal mold. Such high values of the Ra or Rz roughness parameters indicate significant differences between the roughness of the castings. In terms of the casting surface quality, these are limit values. With such surface roughness, it is possible to detect by mere sensitive assessment (touch) considerable differences in the surface quality of the castings made by the wax injection technology and polymer 3D printing.

The roughness of the 3D printed models is significantly affected primarily by the printhead step size, which is, however, often limited by the total printing time of the part and therefore also the production costs. With some materials, it is possible to reduce the model roughness by the smoothing technology using, for example, immersing or steaming in isopropyl alcohol or by penetration of the model with synthetic wax.3.1.3 assessment of thermal expansion of model material.

Thermal expansion of the models is directly connected to the formation of cracks in the ceramic molds. The thermal expansion coefficient is required to be low and the contact pressure of the model on the shell should be lower than the shell strength—MOR (modulus of rapture). The printed models require such infill and structure that allow the model to collapse inwards and thus reduce the contact stress [21].

**Figure 9:**
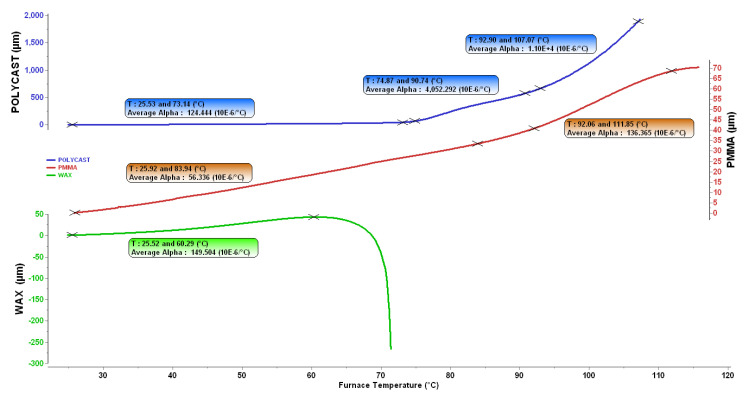
Elongation of samples depending on temperature.

The measurement was made using the Setsys Evolution TMA vertical dilatometer. The test samples are cylinders with a diameter of Ø6 mm and a height of 6 mm (fully filled samples). A temperature gradient of 3 °C/min was used in the measurement and the absolute elongation of the sample was monitored. The measurement was only made for temperatures of up to 120 °C because at higher temperatures the sample stuck to the measuring probe. The measurement was made for the PMMA material (taken directly from the printed BJ model infiltrated with wax) and the A7-FR/1200 model wax used for the injection in a mold technology. A comparison was also made for the PolyCAST™ material, which is a specially developed material (filament-(Polyvinyl butyral)) for FDM model printing and also for the investment casting technology. The elongation values depending on temperature are shown in Figure 9. The curves are evaluated in one figure but, with regard to the order differences in the dilatations measured, the scales are shown separately for each material.

From the values obtained, we can determine the thermal expansion coefficient for each material. The dilatation curves are not linear dependencies in the whole temperature interval and it is therefore necessary to consider the coefficient for a specific temperature interval. Amorphous polymers have a random molecular structure and can gradually soften with increasing temperature. The break temperature is called the glass transition temperature (Tg) [22] and, on the temperature dependent curve, it is the area where a change in the slope (rise) of the curve occurs. Tg is an important characteristic of polymer materials as it represents a point at which a change in the properties of the polymer occurs. More precisely, it is an area, not a specific temperature. Nevertheless, it is usually given as one specific number. Table 2 summarizes the coefficients of thermal expansion and the Tg temperature for each material measured and for specific temperature intervals.

The measurements show that the greatest thermal expansion occurs in the interval up to 60 °C for the model wax. On the contrary, the smallest coefficient in this temperature range belongs to the PMMA material, whose expansion is 2.5 times lower than that of wax. Melting of the model waxes occurs in the temperature range of 60–70 °C, which is apparent as a significant break on the curve around 68 °C. The thermal expansion coefficients of the PMMA and PolyCast™ materials increase at higher temperatures. For PMMA, this increase is not sudden as it is for PolyCast™. The softening temperature for this material given by the producer [23] is 68 °C. From this temperature, the collapse of this material can be assumed. Whether the expansion of the model will cause a rupture in the shell when using the FDM/FFF method depends on the structure and percentage of the filling, air content and, last but not least, shape of the part.

## 4. Discussion

The paper presents a combination of topological optimization and so-called co-design when designing and optimizing an upright for investment casting technology. It was verified that after topological optimization it is necessary to use other virtual tools that will help us solve the manufacturability of the part with the help of hybrid technology. This chaining will not only allow the production of castings on the first try, but in particular significantly shorten the product development cycle. The co-design helped with the adjustment of the wall transitions and pointed out the insufficiently large radii in certain parts of the castings. In reality, this could result in porosity in the casting, or with insufficient radii, it could cause cracks or fissures.

For the hybrid production technology, there is a detailed description of the model production phase and the requirements imposed on the model and its properties. The 3D print technology proved the suitability of the BJ method for making the model. An assessment is made of the model properties primarily with respect to dimensional accuracy, surface quality, and dilatations in the model firing phase. Dimensional changes in the model after printing do not exceed ±0.35 mm, which is compensated for by allowances on the treated surfaces. The Ra roughness values of the PMMA model with an infiltration wax layer do not exceed 12.5 μm. In comparison with the sample injected in a mold (Ra 2.3 μm), this roughness is considerably worse. When measuring the roughness of the infiltrated sample, no significant difference was found in the printing direction or in the transverse printing direction. This is due to the wax’s ability to fill surface irregularities. The ceramic slurry is able to copy any surface irregularities, and to improve the surface roughness of the 3D printed models, it is therefore necessary to pay close attention to pattern post-processing. The issue with wax infiltration is the viscosity of the synthetic wax, the thickness of the layer, and the dripping of the wax so that it does not accumulate in certain parts. The difference found in the roughness of the model produced by wax injection and the printed model is not a problem for prototype parts; however, for small series production in the automotive or aerospace industry, it would be necessary to pay more attention to surface treatment or print settings. The method of coating and its effect on the resulting surface quality will be the subject of further research.

The PMMA material used for the BJ technology shows lower expansion than wax injected in a mold. This property has a positive effect on decreasing the risk of shell cracking during the firing of the model. The better dimensional stability of the PMMA model at room temperatures also allows more optimal transport and storage of the models. The requirements for the model quality were therefore evaluated as suitable. The PolyCAST™ material was included in the measurement only subsequently and it will be necessary to perform additional measurements of thermal expansion with regard to the adhesion of the sample to the measuring probe. This could affect the measurement at the softening temperatures of this material and thus distort the measurement result.

## 5. Conclusions

The work presented the first stage of the hybrid investment casting technology, which is the model production. The work is focused on the design, optimization, and production of the upright model from the PMMA (acrylic plastic) material, which will be used in the next stage for the production of ceramic shells. Based on the investigations carried out in this work, the following conclusions can be drawn:

- Topological optimization of the casting design must be complemented by technological co-design to ensure that the design is technologically feasible.
- Due to its dimensional stability, the PMMA material is more accurate than standard wax models, which makes it possible to eliminate the use of levelling tools for complex shapes.
- The surface quality of the printed model is significantly lower in comparison to wax models produced by injection into metal molds. On the other hand, it still meets the needs of prototype castings. To improve the surface roughness, it would be necessary to increase the print resolution, which would require a change in the 3D printer or the printing technology. Another way is to adjust the infiltration process of the surface layer, either by repeated dipping into a wax suspension, or by adjusting its viscosity.

The production of a ceramic shell and an aluminum prototype casting of the upright is another stage of the work, which will focus on the final comparison of the entire process of hybrid technology. Further studies will be targeted on the evaluation of dimensional accuracy, surface quality, and internal defects in the manufactured metal casting.

## Figures and Tables

**Table 1:** Surface roughness analysis.

Sample A	Sample B
Wax model − metal die	3D printed model − PMMA (binder jetting)
3D model of the sample structure	3D model of the sample structure
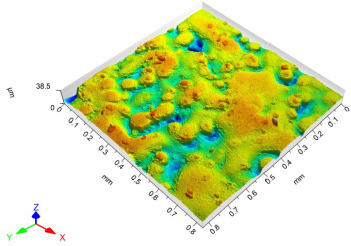	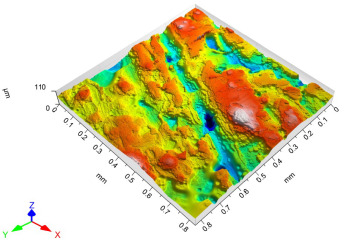
Basic profile along the *X* axis	Basic profile along the *X* axis
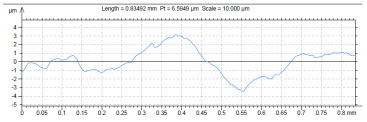	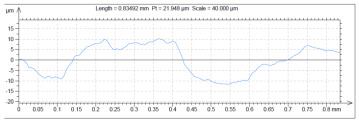
Roughness parameters along the *X* axis	Roughness parameters along the *X* axis
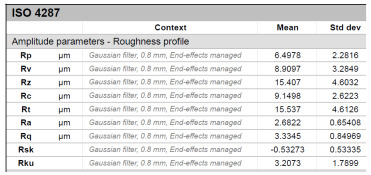	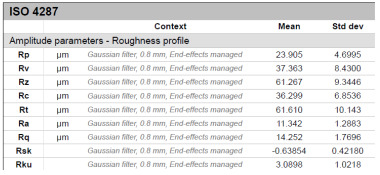
Basic profile along the *Y* axis	Basic profile along the *Y* axis
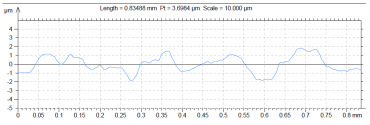	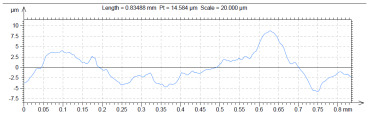
Roughness parameters along the *Y* axis	Roughness parameters along the *Y* axis
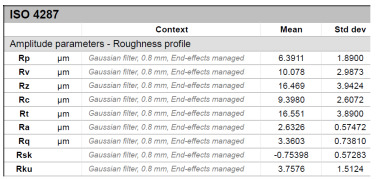	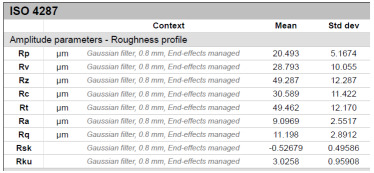

**Table 2:** Comparison of thermal expansion for selected materials.

Material	Temperature Interval [°C]	Thermal Expansion Coeff. [10^−6^/°C]	Tg [°C]
PMMA (BJ)	25.92–83.94	56.34	92
	92.06–111.85	136.37	
WAX	25.52–60.29	149.51	-
Polycast ™ (FDM filament)	25.53–73.14	124.44	75
	74.87–90.74	4052	
	92.9–107.07	11000	
